# DREZotomy in the era of minimally invasive interventions for cancer-related pain management

**DOI:** 10.1097/MS9.0000000000002288

**Published:** 2024-06-17

**Authors:** Areeba Fareed, Malak A. Hassan, Solay Farhat, Afra Sohail, Rayyan Vaid

**Affiliations:** a Karachi Medical and Dental College, Karachi, Pakistan; b Faculty of Medicine, Alexandria University, Alexandria, Egypt; c Lebanese University, Faculty of Medical Sciences, Beirut, Lebanon

## Introduction: understanding cancer-associated pain

Cancer, an intricate and pervasive group of diseases characterized by aberrant and uncontrolled cell division, presents a formidable challenge to individuals, families, and the global healthcare community^1^. Across diverse demographics, cancer manifests in various forms, underscoring the imperative for comprehensive research, prevention, and compassionate care in the ongoing global battle against this relentless adversary. In the field of oncology, where the pursuit of efficacious treatments converges with patient-centric care, cancer pain emerges as a nuanced and multifaceted phenomenon, encapsulating a spectrum of sensations^2^. The progression of cancer gives rise to pain, stemming either from the tumor itself or from the various treatment interventions used, as well as cancer-associated complications. This imposition of pain presents significant challenges for both patients and healthcare providers. Hence, recognition and adept management of cancer pain are pivotal not only for mitigating suffering but also for enhancing the overall well-being of individuals navigating the intricacies of a cancer diagnosis.

The term “cancer pain” encompasses a diverse array of pain conditions, each distinguished by unique origins and characteristics^3^. Primary pain categories comprise neuropathic, somatic, and visceral, with the latter two classified as nociceptive pain linked to harm to body tissues^4^. Duration serves as a defining criterion for pain categorization, encompassing acute, chronic, breakthrough, and refractory pain states^5^. Indeed, cancer-related pain exhibits a high prevalence, impacting 50.7% of patients at any stage and escalating up to 66.4% in advanced stages^6^. Notably, the incidence of pain is ~20% higher in males than females, and the emergence of cancer survivors as a distinct patient group unveils that 27.6% experience moderate-to-severe chronic pain^7^. In fact, a comprehensive meta-analysis conducted by Austin *et al.* ^8^ encompassing 122 studies and 4199 individuals elucidates that ~55% of cancer patients endure pain during therapy, with 40% persisting to experience pain post curative treatment. Diverse factors, including bone metastases and treatment-related side effects such as chemotherapy-induced mucositis, significantly contribute to the complexity of pain experiences in this patient population^9^. Different types of pain management options, including over-the-counter pain relievers and personalized pain control plans, are available to help alleviate cancer pain.

## Management of cancer-related pain: different treatment options

The management of cancer-related pain usually involves a multidisciplinary approach, including medical treatment and non-medical interventions. In order to assess the most appropriate treatment plan, *The European Society for Medical Oncology*’s guide for patients highlights the importance of assessing pain levels using numerical scales, with patients encouraged to report their worst pain as well as average pain^10^. Medical management of neuropathic pain mainly involves medications like tricyclic antidepressants, serotonin and norepinephrine reuptake inhibitors, as well as anticonvulsants, although their use may be accompanied by adverse effects. The combination of these medications with opioids is suggested to enhance pain relief, although specific recommendations are limited^7^. Topical treatments, such as high-concentration capsaicin and lidocaine patches, are recommended for localized neuropathic pain, demonstrating promising results, particularly in cases of chemotherapy-induced peripheral neuropathic pain. Additionally, and in cases of refractory neuropathic pain among patients with a limited prognosis, percutaneous neurolysis emerges as a viable option. Various neurolytic blocks, such as spinal, stellate ganglia, celiac plexus, or splanchnic blocks, are employed based on the location of cancer-related pain. In cases of pain associated with metastatic bone lesions, percutaneous ablation of metastatic lesions using radiofrequency or cryotherapy proves to be a safe and effective method for reducing pain^11^.

## Surgical management of cancer-related pain: DREZotomy

While medical therapy, including opioids, is the mainstay of treatment for cancer pain and is considerably successful in controlling pain in the majority of cases (70–90%), there are instances where pain remains inadequately controlled despite medical interventions^12^. In such cases, a multidisciplinary approach involving non-medical treatments, such as surgical options, may be considered to help manage pain alongside traditional pain medicines. In fact, and although opioids and anticonvulsants constituted conventional modalities for pain management in cancer patients, the advent of side effects and the concomitant risk of dependence prompted the exploration and development of alternative or adjunctive therapeutic strategies^13^. Among these alternatives, interventional techniques, exemplified by peripheral nerve blocks, have gained prominence within the cancer patient population due to their efficacy in the management of severe chronic pain. It is essential to acknowledge, however, that the administration of such procedures may pose challenges, demanding the expertise of skilled personnel and advanced techniques.

**Figure 1:**
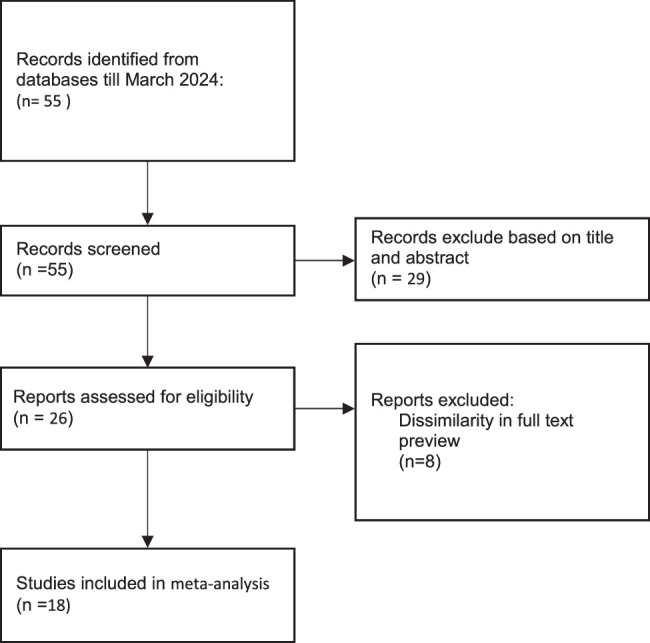
Flowchart to showing studies selection process: PRISMA flow diagram for new systematic reviews which included searches of databases and registers only.

Anatomically, the Dorsal Root Entry Zone (DREZ) constitutes a critical juncture where the dorsal rootlets converge with the dorsolateral fasciculus, known as Lissauer’s tract, situated at the entrance of the dorsal horn of the spinal cord. This region plays a pivotal role in peripheral pain transmission, serving as a key anatomical locus for the integration and modulation of nociceptive signals^14^. The targeting of the DREZ for the regulation of nociception dates back to the 1970s, where the DREZ has garnered attention for its utility in managing well-localized pain across various clinical conditions, including but not limited to instances of brachial plexus avulsion and spinal cord lesions^15^. The application of DREZ-targeted interventions involves precise anatomical localization and modulation of neural pathways to alleviate pain symptoms effectively. While the technique has shown efficacy in managing localized pain in other conditions, its specific application and outcomes in the complex landscape of cancer-related pain warrant further exploration. In fact, advancements in understanding the molecular and cellular mechanisms underlying DREZ-mediated pain modulation may contribute to refining and expanding its therapeutic applications in the field of oncology. Despite the notable potential of DREZ-targeted interventions, scientific literature detailing its application in the context of cancer patients remains relatively limited. The different studies highlighting the potential use of DREZ-targeted interventions in cancer-pain organized in Figure 1.

DREZ lesioning, alternatively known as DREZotomy, represents an emerging strategy for pain management in cancer patients, with various techniques described, including microsurgery, radiofrequency, and laser ablation^15^. The procedure selectively destroys the lateral portions of the dorsal rootlets, which can provide pain relief by lesioning the responsible neurons in the spinal cord segments with avulsed rootlets^16^. While surgical and radiological approaches are more prevalent, a paucity of data precludes conclusive statements regarding the superiority of any specific technique^15^. A recent systematic review evaluating the long-term outcomes of DREZotomy reported “good” outcomes in 98 out of 130 oncologic pain patients, indicating a high level of efficacy^17^. Ruiz-Juretschke *et al.* ^18^ documented pain reduction in 77% of participants undergoing DREZ lesioning via radiofrequency for various conditions, although the three cancer patients in the study experienced either moderate (*n*=1) or no (*n*=2) pain relief.

Indeed, and since DREZotomy is a surgical procedure designed to alleviate chronic pain, particularly neuropathic pain resistant to conventional treatments, it is considered for cancer patients experiencing severe neuropathic pain unresponsive to other interventions. This specialized procedure has shown efficacy in cases such as brachial plexus avulsion, spinal cord injury, and intractable cancer or post-radiation pain^17^. While the decision for DREZotomy is highly individualized, it’s mostly considered as a potential option for cancer types with the closer neurological association, and notably in patients with pancoast tumors^15^. Table 1 shows a detailed dissection of the different studies outcomes seen in the literature.

**Table 1:** 18 included studies with detailed outcomes on DREZotomy

S.no.	Author and year of study	No. patients	Disease/type of cancer diagnosed	Outcomes summary
1.	Nashold, Ostdahl^19^; 1979	21 patients	Avulsion of the cervical dorsal roots of the brachial plexus	13 patients (67%) experienced good pain relief, with follow-up periods ranging from 6 months to 3½ years.
2.	Nashold, Bullitt^20^; 1981	13 patients	Spinal cord injury and paraplegia	Pain relief of 50% or more was achieved in 11 of the 13 patients, with follow-up periods ranging from 5 to 38 months.
3.	Samii, Moringlane^21^; 1984	35 patients	Brachial plexus avulsion, postamputation phantom limb pain, injury to the spine and spinal cord, peripheral nerve lesions, and multiple sclerosis	Satisfactory and long-lasting results, independent of etiology, duration of the pain syndrome, and the quality and projection of the pain.
4.	Saris, Iacono, Nashold^22^; 1985	22 patients	Amputations pain due to trauma, gangrene, or cancer	Overall, 8 (36%) patients experienced complete pain relief, good results were obtained in patients with phantom pain alone or with traumatic amputations associated with root avulsion. Poor results were observed in patients with both phantom and stump pain, or stump pain alone.
5.	Friedman, Nashold^23^; 1986	56 patients	Spinal cord injury	50% of patients experienced good pain relief. Patients experiencing pain extending caudally from the level of the injury and unilateral pain being most likely to obtain relief. Conversely, diffuse pain and predominant sacral pain did not respond as well to the procedure.
6.	Powers, Barbaro, Levy^24^; 1988	40 patients	Various types of deafferentation pain	Good long-term pain relief was observed in some paraplegics and in all patients with brachial plexus avulsion. However, pain associated with arachnoiditis and peripheral nerve injury or neuropathy did not respond
7.	Esposito, Delitala, Nardi^25^; 1988	22 patients	12 patients with Pancoast’s syndrome, 8 patients with cancer pain projected to anesthetic areas, and 2 patients with benign post-thoracotomy pain	For the 12 patients with Pancoast’s syndrome, complete relief from the deafferentative aspect of the pain was observed. For the series of 8 patients with cancer pain and 2 patients with benign post-thoracotomy pain, complete relief from pain was achieved.
8.	Kumagai *et al*.^26^; 1992	15 patients	Chronic pain	Subjective pain relief exceeding 70% was achieved around 2 wk after the operations in most patients (13/15), but it decreased in some to 30% from 70% in follow-up observations. Complications included sensory loss in 12 patients, motor weakness in 7 patients, paresthesia in 4 patients, and new pain in 6 patients. Postmortem histological findings of the spinal cord in two patients with systemic lupus erythematosus and uterine cancer revealed complications, indicating the importance of avoiding extension of coagulation beyond the dorsal horn.
9.	Zeidman, Rossitch, Nashold^27^; 1993	2 patients	RBP	Following DREZ lesions, both patients experienced excellent pain relief immediately postoperatively and remained pain-free at 29–48-month follow-up observations.
10.	Sindou^28^; 1995	367 patients	Cancer pain in 81 patients, neurogenic pain in 139 patients, hyperspasticity in 135 patients, and hyperactive neurogenic bladder in 12 patients	Good outcomes
11.	Kanpolat *et al*.^29^; 2008	55 patients	Various chronic pain conditions that did not respond to medical therapy or other surgical methods, including traumatic brachial plexus avulsions, segmental pain after spinal cord injury, postherpetic neuralgia, topographically limited cancer pain, and atypical facial pain.	Initial success rates for spinal and NC DREZotomy procedures were 77% and 72.5%, respectively. Complications included mortality in one patient in each group, transient muscle weakness and cerebrospinal fluid fistulae in the spinal DREZotomy group, and transient ataxia and transient hemiparesis in the NC DREZotomy group.
12.	Rath *et al*.^30^; 1996	51 patients	Pain due to cervical root avulsion, paraplegia-related pain, postherpetic neuralgia, painful states due to radiation-induced brachial plexopathy, previous surgery, and malignant tumor infiltration of the brachial plexus	For pain due to cervical root avulsion, 77% of patients had permanently good or fair pain relief after a mean follow-up period of 76 months. For paraplegics, 55% experienced continuing pain relief after a mean observation time of 54 months. Poor results were observed, especially in cases of associated spinal cord cysts and in patients with diffuse pain distribution. Continuous marked improvement for longer periods was reported in a small percentage of patients with postherpetic neuralgia and painful states due to radiation-induced brachial plexopathy, previous surgery, and malignant tumor infiltration of the brachial plexus. Major complications, including permanent gait disturbances, were observed in 12% of patients following primary procedures and in a smaller percentage after re-operations. Minor neurological deficits were noted in 18% of cases.
13.	Ruiz-Juretschke *et al*.^18^; 2011	18 patients	Refractory deafferentation pain secondary to spinal cord injury, brachial plexus avulsion, and other peripheral nerve injuries	Pain on the Visual Analog Scale (VAS) significantly decreased from 8.6 preoperatively to 2.9 at discharge (*P*<0.001). Long-term pain remained at 4.7 on the VAS with a mean follow-up of 28 mo (*P*<0.002). The percentage of patients with moderate to excellent pain relief was 77% at discharge and 68% at the last follow-up. Pain medication was reduced in 67% of patients, and 28% returned to work.
14.	Iglesias *et al*.^31^; 2023	3 patients	Oncologic pain, predominantly neuropathic, in pediatric patients	DREZotomy was successfully performed in 3 pediatric patients at the terminal stage of illness, with predominantly neuropathic oncologic pain.
15.	Mazzucchi *et al*.^32^; 2020	2 patients	One patient had tumor in the right pulmonary apex invading the brachial plexus and 2nd patient had pseudomyxoma peritonei with invasion of the right lumbosacral plexus	Hypoesthesia in the targeted area, a decrease in pain, maintenance of the effect at follow-up visits, and a majority of patients experiencing a 75% decrease in pain up to 48 months postoperatively.
16.	Montalvo Afonso *et al*.^33^; 2021	27 patients	The main cause of pain was BPI in 55.6% of patients, followed by neoplasms in 18.5%.	Favorable outcome (≥50% pain reduction in the VAS) was observed in 77.8% of patients. After an average follow-up of 22 mo, the favorable outcome remained in 59.3% of patients, with a mean reduction of 4.9 points on the VAS. Reduction in routine analgesic treatment in 70.4% of patients. DREZotomy for BPI-related pain presented a significantly higher success rate (93%) compared to other pathologies (41.7%).
17.	Piyawattanameth *et al*.^34^; 2017	40 patients	Intractable pain caused by 27 BPIs, 6 spinal cord injuries, 3 neoplasms, and 4 other causes	Significant reduction in both average and maximal pain (*P* <0.001). A favorable outcome (≥50% pain reduction) was observed in 67.5% of patients, with the best outcome observed in BPI-related pain.
18.	Harsh, Viswanathan^35^; 2013	651	Various types of cancer including brachial plexus metastases, Pancoast tumors, thoracic and abdominal wall invasion, lumbosacral nerve involvement, and metastatic cancer	Significant pain relief in a high percentage of patients, with some complications generally manageable

BPIs, brachial plexus injuries; DREZ, Dorsal Root Entry Zone; RBP, Radiation-induced brachial plexopathy.

Additionally, and while DREZotomy has successfully showed promises in cancer-related pain management, it is not without significant complications, the most common being neurological deficits of varying severity^17,33^. Although post-operative infections and procedure-related deaths have been reported as well, they are considered as infrequent occurrences^17^. A retrospective study involving 27 patients undergoing DREZotomy for pain from various pathologies revealed six neurological complications, with one-third being irreversible^33^. Notably, limited data exists on complications in patients with oncologic pain. Interestingly, DREZotomy lesioning demonstrates greater success in brachial plexus avulsion compared to other pathological causes of pain, a trend potentially influenced by the uneven representation of various DREZotomy indications in the published literature and as is shown in Table 1. This observation underscores the potential significance of patient selection and population-related factors. Indeed, exploring the feasibility of implementing DREZotomy in healthcare institutions reveals potential benefits in managing chronic neuropathic pain. The targeted approach of DREZotomy offers a promising avenue for pain relief, potentially reducing long-term medication dependence and improving patients’ overall quality of life. However, challenges such as the need for specialized surgical expertise, precise patient selection criteria, ethical considerations, and resource allocation must be carefully navigated. Performing DREZotomy necessitates specific surgical proficiency and expertise.

Lastly, one last factor to consider is the feasibility and cost-effectiveness. DREZotomy requires a careful examination of both immediate costs and long-term results. Although the initial expenses linked to specialized surgical procedures and postoperative care may be significant, the possibility of sustained pain relief and enhanced patient quality of life holds the potential for long-term financial benefits. This is attributed to a diminished reliance on extensive medication management and subsequent healthcare interventions. The current body of evidence on DREZotomy outcomes remains inconclusive, primarily comprised of case series with variations in lesioning techniques, cancer types, and population characteristics contributing to the lack of definitive conclusions^15^. Despite these challenges, the compelling need for further research persists, given the promising results and potential benefits of DREZotomy in managing oncologic pain.

## Limitations of DREZotomy

DREZotomy presents a promising approach for managing cancer-related pain, albeit with notable complications. Neurological deficits, though common, can vary in severity, with some being irreversible. While postoperative infections and deaths are rare, they do occur. Limited data exist on complications specific to oncologic pain, which highlights the need of more research studies focusing primarily on cancer patients with chronic pain. Despite its potential benefits in chronic neuropathic pain management, challenges such as surgical expertise, patient selection, and cost-effectiveness remain. Further research is crucial to better understand its outcomes and potential in oncologic pain management.

## Conclusion

In conclusion, the pervasive and complex nature of cancer necessitates an ongoing commitment to comprehensive research, prevention, and compassionate care. Cancer pain, as a multifaceted phenomenon, poses significant challenges for patients and healthcare providers alike, demanding adept recognition and management for improved well-being. The spectrum of cancer-related pain, categorized by diverse origins and characteristics, underscores the need for innovative approaches to address this critical aspect of the disease. From conventional pharmacological interventions to emerging techniques like Dorsal Root Entry Zone (DREZ) lesioning, the landscape of pain management in cancer patients is evolving. While the efficacy of DREZotomy shows promise, its application in oncologic pain warrants further exploration, considering the limited literature available. Notably, success in brachial plexus avulsion highlights the potential influence of patient selection and population-related factors. Perhaps it is time to further establish and document the safety and efficacy of DREZotomy in cancer pain through large-scale trials in various populations and settings. In doing so, we may witness a shift in oncologic pain management recommendations in the coming years. With its high potential effectiveness, DREZ lesioning offers a glimpse of hope for patients who have had their quality of life significantly diminished by malignancy-related pain. Despite challenges in interpreting outcomes, the compelling need for continued research in this field is evident, driven by the promising results and potential benefits that innovative strategies like DREZotomy may offer in the complex landscape of oncologic pain management.

## Ethical approval

Not applicable.

## Consent

Not applicable.

## Source of funding

Not applicable.

## Author contribution

All authors have contributed equally to this work.

## Conflicts of interest disclosure

The authors declare no conflicts of interest.

## Research registration unique identifying number (UIN)

Not applicable.

## Guarantor

Solay Farhat.

## Data availability statement

Not applicable.

## Provenance and peer review

Not invited.
